# Asymmetric coordinate attention spectral-spatial feature fusion network for hyperspectral image classification

**DOI:** 10.1038/s41598-021-97029-5

**Published:** 2021-08-31

**Authors:** Shuli Cheng, Liejun Wang, Anyu Du

**Affiliations:** College of Information Science and Engineering, Xinjiang University, Urumqi, 830046 China

**Keywords:** Engineering, Physics

## Abstract

In recent years, the hyperspectral classification algorithm based on deep learning has received widespread attention, but the existing network models have higher model complexity and require more time consumption. In order to further improve the accuracy of hyperspectral image classification and reduce model complexity, this paper proposes an asymmetric coordinate attention spectral-spatial feature fusion network (ACAS2F2N) to capture distinguishing hyperspectral features. Specifically, adaptive asymmetric iterative attention was proposed to obtain the discriminative spectral-spatial features. Different from the common feature fusion method, this feature fusion method can adapt to most skip connection tasks. In addition, there is no manual parameter setting. Coordinate attention is used to obtain accurate coordinate information and channel relationship. The strip pooling module was introduced to increase the network’s receptive field and avoid irrelevant information brought by conventional convolution kernels. The proposed algorithm is tested on the mainstream hyperspectral datasets (IP, KSC, and Botswana), experimental results show that the proposed ACAS2F2N can achieve state-of-the-art performance with lower time complexity.

## Introduction

Hyperspectral image data has rich spectral-spatial information, and hyperspectral image classification tasks have important research significance, such as crop detection, geological prospecting and other fields^1^. However, the inherent characteristics of hyperspectral data (spatial pixel non-uniformity, spectral noise and frequency band correlation) affect the performance of hyperspectral image classification to a certain extent. Make full use of hyperspectral-spatial data to improve the performance of hyperspectral image classification, which has become a research hotspot in hyperspectral data analysis and processing^2–5^. At the same time, the processing methods of hyperspectral image classification tasks include traditional methods^6,7^ and deep learning methods^8,9^.

In recent years, deep learning-based methods have become the mainstream algorithm for hyperspectral image classification. Specifically, in terms of spectral-spatial feature extraction, Roy et al.^2^ proposed a residual squeeze-and-excitation to extract joint spectral-spatial features. Tang et al.^3^ introduced octave convolution to capture features with strong discriminative ability. Tang et al.^4^ developed an improved residual network, which has a spectral-spatial kernel function and receptive field of different scales (A2S2K-ResNet). Xu et al.^5^ studied the fully convolutional network based on dense conditional random fields and atrous convolution to deal with hyperspectral classification. Roy et al.^8^ explored hybrid spectral CNN (HybridSN) by combining 3D CNN and 2D CNN. Guo et al.^9^ proposed feature-grouped network, the method includes spatial residual block and spectral residual block. He et al.^10^ used transfer learning to obtain hyperspectral image representation. Gao et al.^11^ explored the multi-scale residual network based on mixed convolution to obtain fusion features. Lu et al.^12^ presented a multi-scale residual network based on channel and spatial attention to perform hyperspectral image classification. Li et al.^13^ proposed a double-branch dual-attention (DBDA), which includes spectral attention and spatial attention. Zhong et al.^14^ explored the spectral–spatial residual network (SSRN) based on 3D convolutional layers. Wang et al.^15^ studied fast dense spectral–spatial convolution (FDSSC), which is a densely-connected structure to learn hyperspectral features. Ge et al. merged 2D CNN and 3D CNN, and proposed a hyperspectral classification method based on multi-branch feature fusion^16^. Lee et al.^17^ proposed a deeper contextual CNN (ContextNet), which can enrich the spectral-spatial features of hyperspectral. Paoletti et al.^18^ studied the deep pyramidal residual network (DPyResNet), which focuses on the depth of the network block and the generation of more feature maps.

The construction of the network model has become the main technology of hyperspectral image classification, and it is also the current research focus of hyperspectral classification. In terms of graph convolutional networks, Mou et al.^19^ proposed a nonlocal graph convolutional network (GCN). Wan et al.^20,21^ explored multi-scale dynamic GCN based on superpixel segmentation of simple linear iterative clustering (SLIC). Yang et al. proposed GCN hyperspectral classification by sampling and aggregating, referred to as GraphSAGE^22^. Liu et al.^23^ studied GCN hyperspectral classification based on label consistency and multi-scale convolutional networks. Ding et al.^24^ proposed a globally consistent GCN based on SLIC. Hong et al. fuse CNN features and GCN features to perform hyperspectral classification^25^. Sha et al. proposed Graph Attention Network based on KNN and attention layer^26^. Zhao et al. explored the spectral-spatial GAT to reserve the discriminative features of hyperspectral^27^. In short, GCN plays an important role in the classification of hyperspectral images.

Summary of current research: For hyperspectral classification tasks, researchers have explored many classic algorithms, and these studies have further improved the accuracy of hyperspectral image classification. The relevant research summary is described as follows:

1. *Network model* Residual networks and densely connected networks are the main network types for current hyperspectral classification. The fusion of 2D CNN and 3D CNN is also a trend in hyperspectral research. Inserting the network module into the backbone network (such as the residual network) may improve the performance of hyperspectral classification. However, it will increase model parameters and consume time complexity.
2. *Multi-feature fusion* Hyperspectral image data has rich spectral-spatial features, so multi-feature fusion is the main method of hyperspectral classification. Related technologies include: multi-scale fusion, multi-branch fusion, spectral-spatial fusion, etc. The adaptability and effectiveness of feature fusion still need to be considered, which can avoid manual parameter adjustment and improve the accuracy of hyperspectral classification.
3. *Attention mechanism* The attention mechanism can effectively enhance hyperspectral features and select discriminative spectral-spatial features. In computer vision, most of the existing attention mechanisms have high complexity, and there is less exploration of low-rank attention networks. This is also very important to expand the receptive field of the network and choose a suitable attention model.

### The main contributions of this paper

Compared with most existing hyperspectral classification baselines, in order to further improve the accuracy of hyperspectral image classification and reduce model complexity, this paper proposes a new network model to deal with hyperspectral classification tasks. The algorithm is called ACAS2F2N. The main contributions of this paper are summarized as follows:

1. This paper proposes an asymmetric coordinate attention spectral-spatial feature fusion network (ACAS2F2N) to complete the hyperspectral classification task. ACAS2F2N is an asymmetric learning model, and it is an end-to-end feature learning method. The proposed algorithm can improve the performance of hyperspectral image classification and has lower model complexity.
2. The adaptive iterative attention feature fusion method is adopted to extract the discriminative spectral-spatial features. Different from the common feature fusion method, this feature fusion method can adapt to most skip connection tasks. In addition, there is no manual parameter setting.
3. Coordinate attention is used to obtain accurate coordinate information and channel relationship. The strip pooling module was introduced to increase the network’s receptive field and avoid irrelevant information brought by conventional convolution kernels.
4. The proposed algorithm is tested on the mainstream hyperspectral datasets (IP, KSC, and Botswana), experimental results show that the proposed ACAS2F2N can achieve state-of-the-art performance with lower time complexity.

The rest of the paper are organized as follows. “Related work” section describes the proposed ACAS2F2N network architecture. “ACAS2F2N network architecture” section shows the experimental results. “Results” section elaborates the conclusion.

## Related work

In hyperspectral classification tasks, generative adversarial network^28–30^, long short-term memory^31^, network architecture search^32^, and capsule network^33–35^ are all used in hyperspectral classification. In addition, Hao et al. proposed a hyperspectral classification algorithm based on recurrent neural network and geometry-aware loss, referred to as Geo-DRNN^36^. In the attention mechanism, Xue et al. proposed a second-order covariance pooling network based on attention, which reduces model complexity while ensuring classification accuracy^37^. Gao et al. combined 2D CNN and 3D CNN to construct a deep covariance attention network^38^. Gao et al. proposed densely connected multiscale attention network by combining multi-scale technology and densely connected structure^39^. Hang et al. explored attention-aided CNN based on the spectral attention module and the spatial attention module, and completed hyperspectral classification based on feature fusion^40^. Li et al.^41^ proposed a multi-attention fusion network by fusing the band attention module and the spatial attention module. Xi et al. extracted multi-stream hyperspectral features, integrated corresponding features, and introduced a fully connected layer to perform hyperspectral classification^42^. Xi et al. explored hybrid residual attention. In the feature extraction process, 3D CNN features, 2D CNN features, and 1D CNN features are acquired to make corresponding decisions^43^. The attention mechanism is also a key technology to improve the performance of hyperspectral classification.

The construction of the network model is still the main technology of hyperspectral classification. Specifically, Sun et al. proposed^44^ a fully convolutional segmentation network, and its core structure is still a residual block. Pan et al.^45^ presented a semantic segmentation network to reduce the adjustment of manual parameters of the model. Transfer learning is also one of the research contents of hyperspectral feature extraction^46,47^. GhostNet module and channel attention are selected to improve the accuracy of hyperspectral classification^48^. A nonlocal module and a fully convolutional network were chosen to improve the effectiveness of hyperspectral classification^49^. Li et al. proposed a multi-layer fusion dense network based on the 3D dense module to extract spectral-spatial features^50^. Xie et al. proposed a multiscale densely-connected fusion network based on the multi-scale fusion and dense module for hyperspectral image classification^51^. Meng et al. developed a mixed link network based on addition, concatenation and dense modules^52^. Wei et al. proposed an unsupervised feature learning method, and the loss function is based on classification loss and clustering loss^53^. Zhang et al. fuse low-level residual blocks, medium-level residual blocks and high-level residual blocks to build a deep feature integration network^54^. Li et al. explored a two-stream convolutional network to obtain global and local features^55^. Yuan et al. proposed a proxy-based deep learning framework to learn Hyperspectral features^56^. Zheng et al. mixed 2D CNN and 3D CNN, and introduced covariance pooling to reduce the channel dimension^57^. Jiang et al. carried out related research on hyperspectral classification in spatial consistence^58^. Mu et al. separated low-rank components and sparse components, and implemented a two-branch network^59^. The convolutional layer and residual module still play an important role in hyperspectral classification^60–64^. In addition, support vector machines^65^, self-learning^66^, multi-view feature^67^ are used to obtain discriminative features to perform hyperspectral classification. Some methods based on RNN and LSTM are also widely used in the field of histopathology^68–70^. The goal of these methods is to obtain highly discriminative spectral-spatial fusion features. In short, hyperspectral classification has become a research hotspot in the field of remote sensing and related research is of great significance.

## ACAS2F2N network architecture

### Research motivation

At present, there are still some problems in hyperspectral classification that need to be dealt with: (1) hyperspectral images have rich spectral and spatial information. The spatial and spectral information of hyperspectral images are not fully utilized; (2) most existing hyperspectral classifications have high model complexity. In other words, the network model has more parameters.

Although many algorithms based on attention mechanism have been proposed in the field of computer vision, if the attention mechanism is directly inserted into the backbone network, the time complexity of the algorithm will inevitably increase. In addition, directly fusing two independent networks will also increase algorithm time complexity and model complexity. Based on the above considerations, the goal of this paper is to independently build a hyperspectral classification network. The proposed network can achieve better hyperspectral classification performance with lower model complexity.

### Algorithm advantages

In order to further improve the accuracy of hyperspectral image classification and reduce the time loss of hyperspectral classification, this paper proposes asymmetric coordinate attention spectral-spatial feature fusion network (ACAS2F2N). The proposed algorithm has the following major advantages:

1. Different from most existing algorithms, this paper does not use residual network and dense connection module to obtain hyperspectral image features, so the proposed algorithm does not increase the model complexity.
2. Coordinate attention is used to extract discriminative spectral-spatial features. Different from existing hyperspectral classification algorithms, coordinate attention can accurately obtain coordinate information and channel relationships. In addition, coordinate attention is also proposed for the first time in hyperspectral processing.
3. Strip pooling increases the network receptive field and optimizes the feature map. Strip kernel function can avoid the introduction of irrelevant information.
4. Asymmetric iterative attention feature fusion can obtain multi-scale information. This feature fusion method conveniently uses skip connection information, so it has better scalability. In addition, there is no manual parameter setting.

#### ACAS2F2N overall architecture

**Figure 1:**
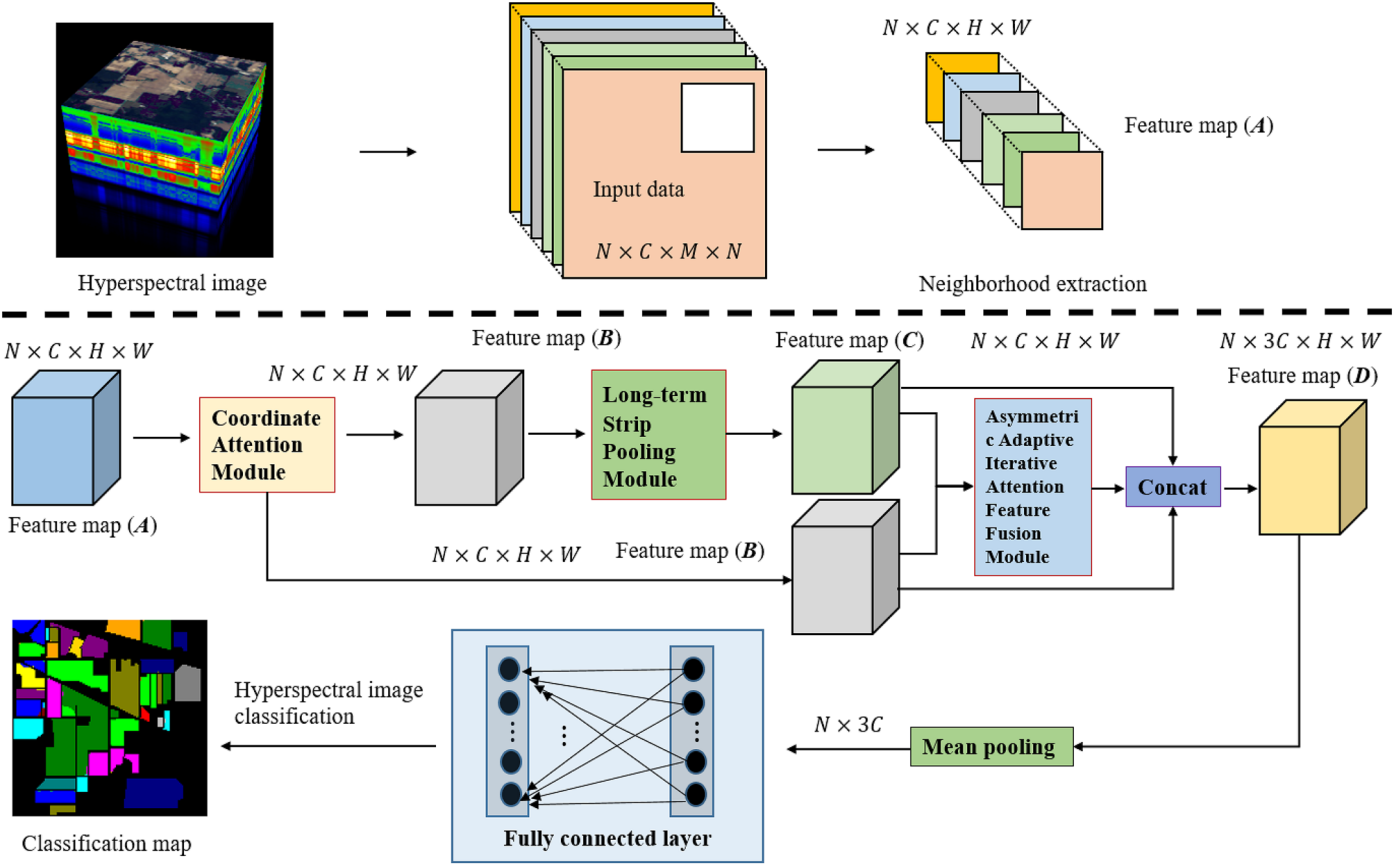
The overall architecture of the proposed ACAS2F2N.

The overall architecture of the proposed ACAS2F2N is shown in Fig. 1. The goal of ACAS2F2N is to complete the classification of hyperspectral images and reduce the time consumption of the model. In Fig. 1, the basic steps of ACAS2F2N are as follows: (1) obtain the feature map (A). Specifically, for a given input hyperspectral data, the size of the hyperspectral image is $C\times M\times N$, the neighborhood feature map (${\varvec{A}}$) is obtained based on neighborhood extraction. The size of feature map (${\varvec{A}}$) is $C\times M\times N$; (2) coordinate attention is used to accurately capture the position information and channel relationship in the feature map (A). Specifically, the spectral-spatial information in the hyperspectral data is captured based on coordinate attention. After coordinate attention, ACAS2F2N can obtain the feature map (${\varvec{B}}$). The size of the feature map (${\varvec{B}}$) is $C\times M\times N$; (3) establish long-term connections, eliminate irrelevant information and increase the receptive field. The strip pooling module converts feature map (${\varvec{B}}$) to feature map (${\varvec{C}}$). Different from the traditional convolution kernel function, strip pooling adopts a narrow-band kernel to avoid the negative influence of irrelevant information. In addition, the strip pooling module has the ability to establish long-term connections and increase the receptive field of ACAS2F2N; (4) adaptive asymmetric iterative attention feature fusion module (A2IAFFM). The main function of A2IAFFM is to adaptively fuse feature map (${\varvec{B}}$) and feature map (${\varvec{C}}$). The feature map acquisition process is an asymmetric way, so it is named A2IAFFM. Through A2IAFFM, ACAS2F2N can obtain spectral-spatial features with discriminative capabilities. In addition, compared to most existing feature fusion modules, A2IAFFM does not require additional manual parameter settings while acquiring multi-scale information. After A2IAFFM, the feature map (${\varvec{D}}$) is obtained, and the size of the feature map is $C\times M\times N$; (5) to complete hyperspectral image classification based on mean pooling and fully connected layer. The size of the mean pooling output is $N\times 3C$. Finally, the hyperspectral classification task is completed through the fully connected layer and cross entropy loss.

The working principle of ACAS2F2N is as follows: (1) obtain feature map (A); (2) coordinate attention is used to obtain accurate coordinate information and channel relationship; (3) strip pooling module was introduced to increase the network’s receptive field and avoid irrelevant information brought by conventional convolution kernels; (4) the adaptive iterative attention feature fusion method is adopted to extract the discriminative spectral-spatial features; (5) complete hyperspectral image classification based on mean pooling and fully connected layers.

In Fig. 1, the definition of symbols is described as follows: $N$ is the batch size, $C$ is the channel size, and $C$ is the hyperspectral band size. $M\times N$ is the size of the hyperspectral image spatial domain, $M$ is the height of the hyperspectral image spatial domain, and $N$ is the width of the hyperspectral image spatial domain. $H\times W$ is the size of the feature map, $H$ is the height of the feature map, and $W$ is the width of the feature map.

#### Coordinate attention module

Existing hyperspectral image classification algorithms, location information (hyperspectral spatial domain) and channel relationship (hyperspectral image frequency band) have received less attention. Hyperspectral images have rich spectral-spatial information, so coordinate attention module^71^ is used in this paper to obtain accurate position information and channel relationships of hyperspectral images.

Channel attention pays less attention to location information, and spatial attention pays less attention to channel relationships. The mixed-domain attention mechanism can consider location information and channel information at the same time. The mixed-domain attention mechanism is widely used in computer vision, most of the existing attention mechanism models have high computational complexity^72,73^. In addition, related studies have shown that low-rank attention and lightweight attention are less studied in computer vision^74,75^.

**Figure 2:**
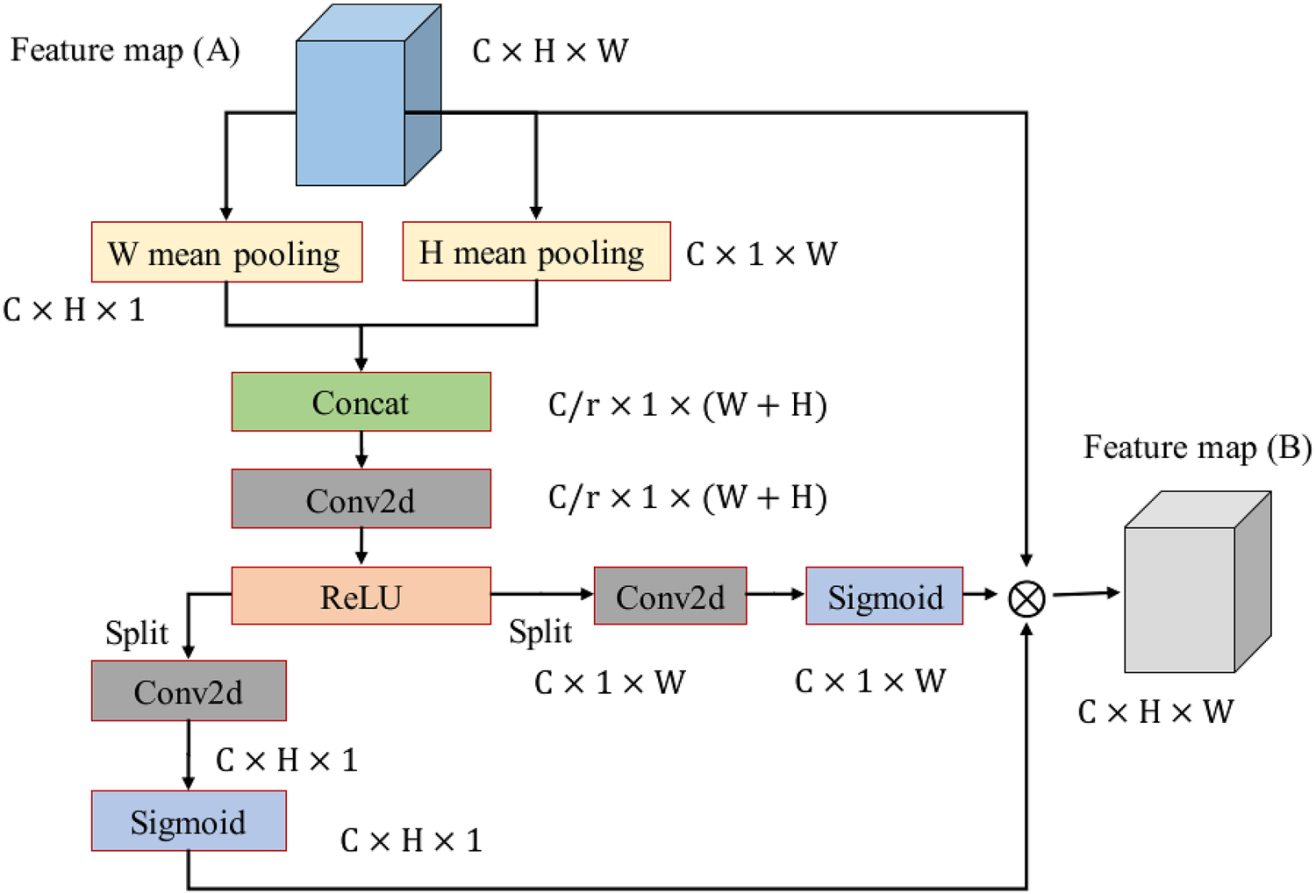
Coordinate attention module. The coordinate attention module can accurately capture the coordinate information of hyperspectral images.

In order to obtain the precise location information of the hyperspectral, we use coordinate attention to extract the feature map (${\varvec{B}}$). The coordinate attention module mainly captures position information, and the structure of the coordinate attention module is shown in Fig. 2.

In Fig. 2, the coordinate attention module only encodes $H$ and $W$. In hyperspectral image, given position $\left(i,j\right)$, the pixel value on channel $c$ is ${x}_{c}\left(i,j\right)$.

The output of $W$ mean pooling is defined as follows:

1 $${y}_{c}^{i}\left(i\right)=\frac{1}{W}{\sum }_{0\le j\le \mathrm{W}}{x}_{c}\left(i,j\right).$$

The output of $H$ mean pooling is defined as follows:

2 $${y}_{c}^{j}\left(j\right)=\frac{1}{H}{\sum }_{0\le j\le \mathrm{H}}{x}_{c}\left(i,j\right).$$

In Fig. 2, the coordinate attention module then completes the concatenate, convolution, and activation function operations. The relevant definitions are as follows:

3 $$y=\delta \left(F\left(\left[{y}_{c}^{i},{y}_{c}^{j}\right]\right)\right),$$

where $\left[,\right]$ is concatenate, $F$ is $1\times 1$ convolution operation, and $\delta$ is ReLU activation function. $y$ is the output feature map of the ReLU layer.

After the split operation, $y$ can be decomposed into ${y}^{i}$ and ${y}^{j}$. Next, ${y}^{i}$ completes the weighting of $W$ through convolution and activation function. ${y}^{j}$ completes the weighting of $H$ through convolution and activation function. The relevant definitions are as follows:

4 $${w}^{i}=\sigma\left({F}_{i}\left({y}^{i}\right)\right),$$

5 $${w}^{j}=\sigma\left({F}_{j}\left({y}^{j}\right)\right),$$

where ${F}_{i}$ is the convolution operation on $H$, and its input is ${y}^{i}$.

${w}^{i}$ is the adaptive weighting of the $H$ direction of the hyperspectral data. ${F}_{j}$ is the convolution operation on $W$, and its input is ${y}^{j}$. ${w}^{j}$ is the adaptive weighting of the $W$ direction of the hyperspectral data. $\sigma$ is the sigmoid activation function.

The output feature map (${\varvec{B}}$) of the coordinate attention module is defined as follows:

6 $${f}_{c}\left(i,j\right)={x}_{c}\left(i,j\right)\times {w}_{c}^{i}\left(i\right)\times {w}_{c}^{j}\left(j\right),$$

where $c$ is the $c$-th channel. ${w}_{c}^{i}\left(i\right)$ is the weight of the $i$-th position in the H direction. ${w}_{c}^{j}\left(j\right)$ is the weight of the $j$-th position in the W direction. Given position $\left(i,j\right)$, ${x}_{c}\left(i,j\right)$ is the value of the input feature map (${\varvec{A}}$). ${f}_{c}\left(i,j\right)$ is the value of the output feature map (${\varvec{B}}$).

#### Long-term strip pooling module

**Figure 3:**
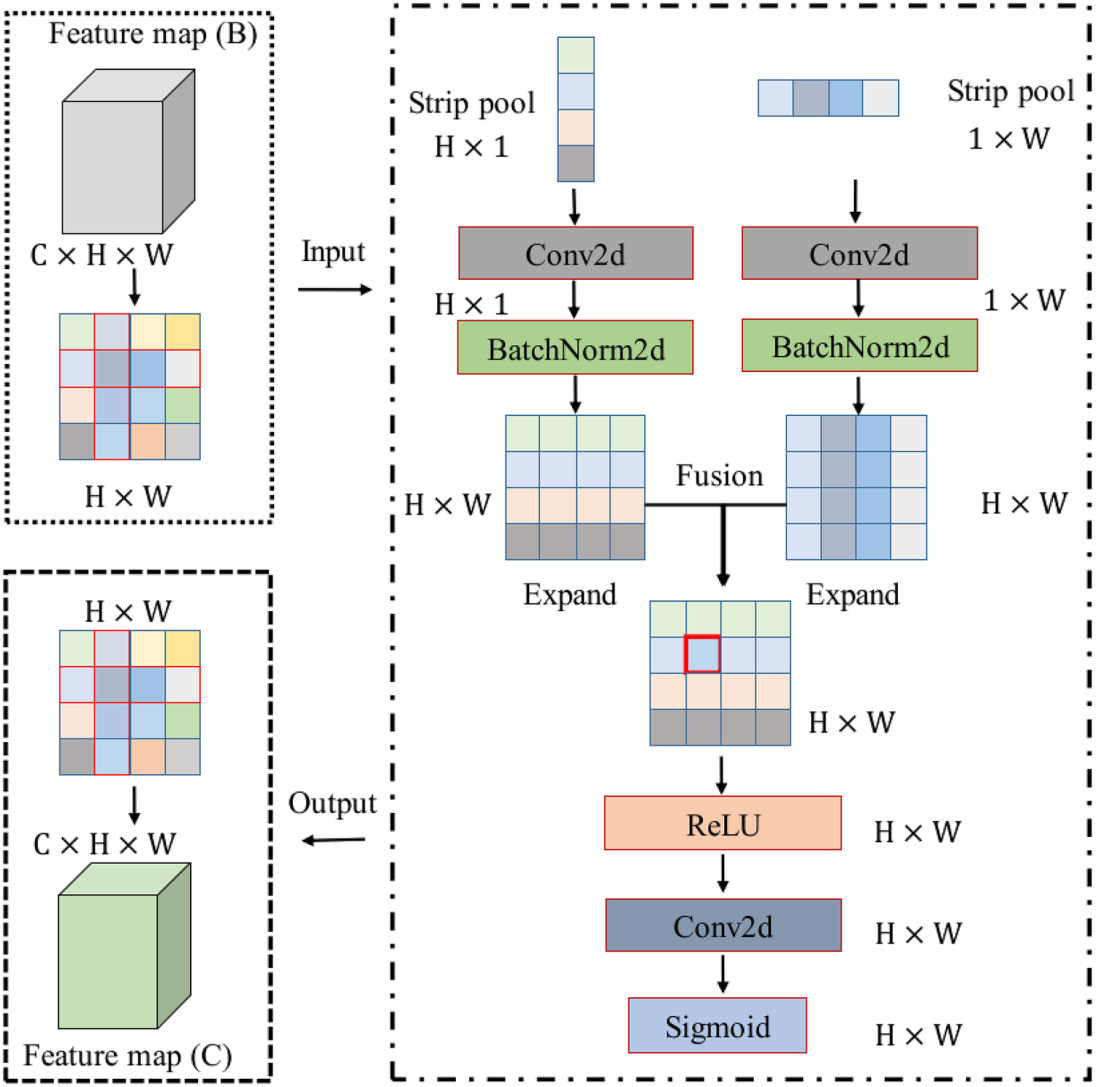
Long-term strip pooling module. The narrowband kernel function is used to expand the network receptive field. Compared with the traditional kernel function, the strip pooling module can better capture the region information of the hyperspectral image.

Different from the traditional kernel function, the strip pooling module uses a narrow-band kernel function to enhance the network receptive field. In addition, the strip pooling module can obtain long-term hyperspectral information and avoid the influence of negative information in hyperspectral images. In Fig. 3, strip pooling uses a narrow-band kernel function to focus on the regional information of the hyperspectral image.

The input of the long-term strip pooling module is the feature map (${\varvec{B}}$), namely ${f}_{c}\left(i,j\right)$. The definition of strip pooling is as follows:

The output of W strip pooling is defined as follows:

7 $${g}_{c}^{i}\left(i\right)=\frac{1}{\mathrm{W}}{\sum }_{0\le j\le \mathrm{W}}{f}_{c}\left(i,j\right).$$

The output of H strip pooling is defined as follows:

8 $${g}_{c}^{j}\left(j\right)=\frac{1}{\mathrm{H}}{\sum }_{0\le j\le \mathrm{H}}{f}_{c}\left(i,j\right).$$

Next, the strip pooling module executes Conv2d and Batchnorm2d. The relevant definitions are as follows:

9 $${h}^{i}=BN\left({M}_{i}\left({g}_{c}^{i}\left(i\right)\right)\right),$$

10 $${h}^{j}=BN\left({M}_{j}\left({g}_{c}^{j}\left(j\right)\right)\right),$$

where ${M}_{i}$ is the conv2d operation, and its kernel function size is $1\times W$. ${M}_{j}$ is the Conv2d operation, and its kernel function size is $H\times 1$. $BN$ is a Batchnorm2d operation. ${h}^{i}$ and ${h}^{j}$ are the output of the Batchnorm2d layer, respectively. $c$ is the $c$-th channel.

The related definitions of feature fusion, ReLU, Conv2d, and activation function are as follows:

11 $$N=\sigma\left({f}_{2}\left({{f}_{1}(h}^{i}+{h}^{j})\right)\right),$$

where ${f}_{1}$ is the ReLU operation, ${f}_{2}$ is the Conv2d operation, and $\sigma$ is the sigmoid function. $N$ is the feature map (${\varvec{C}}$), and $N$ is also the output of the long-term strip pooling module.

#### Asymmetric adaptive iterative attention feature fusion module (A2IAFFM)

**Figure 4:**
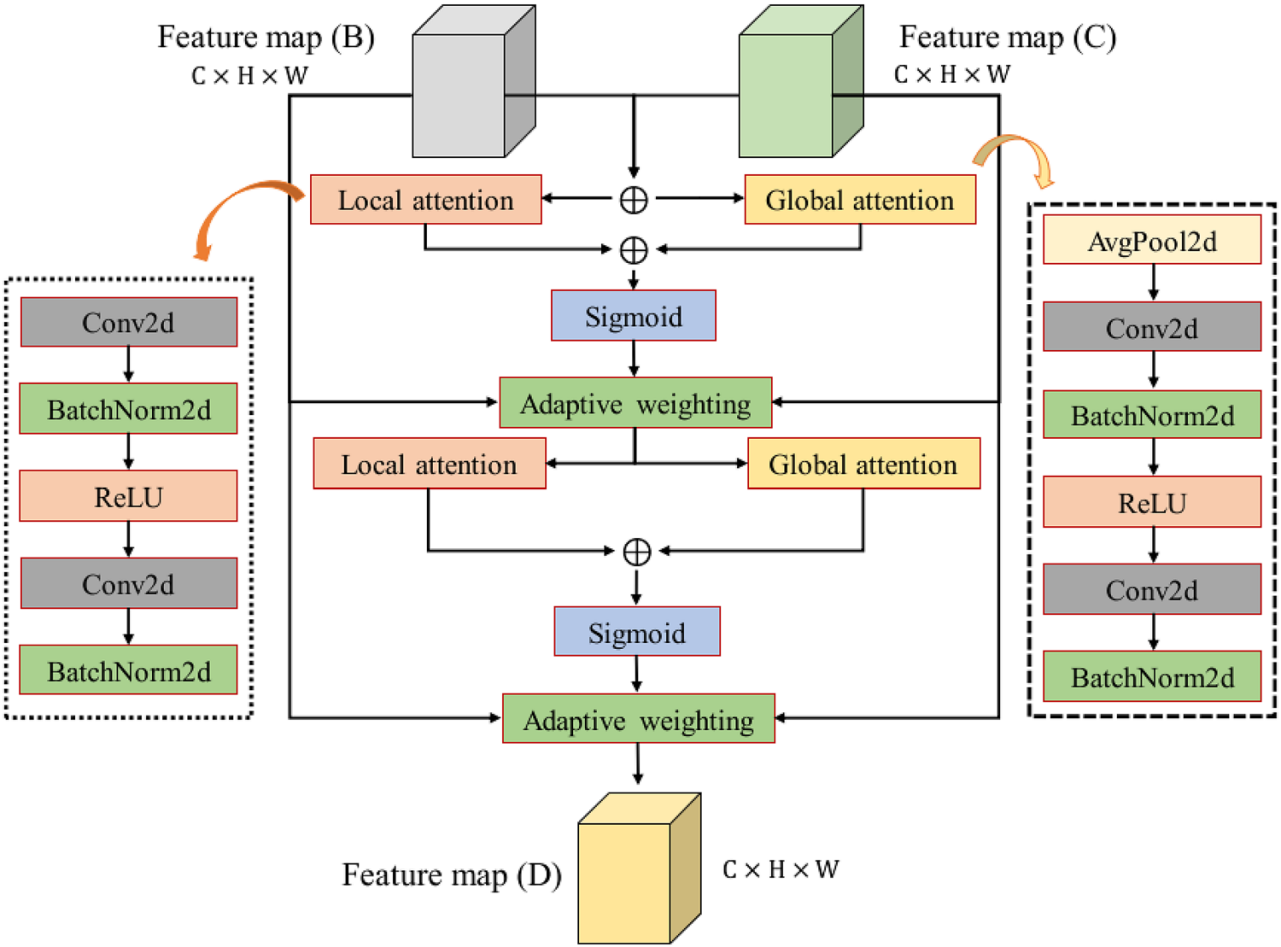
Asymmetric adaptive iterative attention feature fusion module. The input feature maps are B and C, and the output feature map is $\mathrm{D}$.

In Fig. 4, The goal of A2IAFFM is to complete the fusion of feature map (${\varvec{B}}$) and feature map (${\varvec{C}}$). A2IAFFM can acquire hyperspectral features with discriminative ability. The output of A2IAFFM is the feature map (${\varvec{D}}$). A2IAFFM includes local attention, global attention and adaptive weighting.

To simplify the description, $L$ represents the local attention function, and $G$ represents the global attention function. $\sigma$ is the sigmoid function. The adaptive weighting is defined as follows:

12 $$Z=W\otimes X+\left(1-W\right)\otimes Y,$$

where $W$ is the output weight of sigmoid function, and $X$ and $Y$ are the two input feature maps. $\otimes$ is element-wise product.

The first weight calculation process of A2IAFFM is defined as follows:

13 $${W}_{1}=\sigma\left(L\left({\varvec{B}}+{\varvec{C}}\right)+G\left({\varvec{B}}+{\varvec{C}}\right)\right),$$

where the input feature maps are B and C, and the output is ${W}_{1}$.

The A2IAFFM first adaptive weighting calculation process is defined as follows:

14 $${Z}_{1}={W}_{1}\otimes {\varvec{B}}+\left(1-{W}_{1}\right)\otimes {\varvec{C}}.$$

The second weight calculation process of A2IAFFM is defined as follows:

15 $${W}_{2}=\sigma\left(L\left({Z}_{1}\right)+G\left({Z}_{1}\right)\right).$$

The A2IAFFM second adaptive weighting calculation process is defined as follows:

16 $${Z}_{2}={W}_{2}\otimes {\varvec{B}}+\left(1-{W}_{2}\right)\otimes {\varvec{C}}.$$

In formula (16), the output feature map (D) of A2IAFFM can be obtained, denoted by ${Z}_{2}$.

#### Other related technical modules

As shown in Fig. 1, other related technology modules mainly explain the details of the mean pooling layer and the fully connected layer. Among them, the average pooling layer is based on the AdaptiveAvgPool2d operation. There are two fully connected layers. The number of input nodes is the number of frequency bands of the feature map, and the number of output nodes is the number of categories of hyperspectral classification. In addition, this paper uses cross-entropy loss to optimize the network parameters of proposed ACAS2F2N.

#### Parameter configuration

Figure 1 shows the overall architecture of the proposed ACAS2F2N, while Figs. 2, 3 and 4 show the network structures of the coordinate attention module, strip pooling module, and A2IAFFM, respectively. Next, the parameter configuration of the proposed algorithm is shown in Table 1. In the coordinate attention module, its core contains 2 mean pooling layers ($W$ mean pooling and $H$ mean pooling) and 3 convolutional layers (Conv2d). In the strip pooling module, the module contains a total of 3 convolutional layers, namely ConvH, ConvW and Conv2d. In A2IAFFM, the module contains 8 convolutional layers (Conv2d). The three modules coordinate attention module, strip pooling module and A2IAFFM obtain the hyperspectral feature map (${\varvec{D}}$) through concat operation. At this time, the number of channels of ${\varvec{D}}$ is $3C$. Therefore, the input of the fully connected layer (FC) is *3C*, and the output of the fully connected layer (FC) is the number of categories.

**Table 1:** Parameter configuration of the proposed network.

Network module	Network layer	Parameter configuration
Coordinate attention module	$W$ mean pooling	Output_size = (1, 9)
	$H$ mean pooling	Output_size = (9, 1)
	Conv2d (3 layers)	Kernel_size = (1, 1), stride = (1, 1)
Strip pooling module	ConvH (Conv2d)	Kernel_size = (3, 1), stride = (1, 1), padding = (1, 0)
	ConvW (Conv2d)	Kernel_size = (1, 3), stride = (1, 1), padding = (0, 1)
	Conv2d (1 layers)	Kernel_size = (1, 1), stride = (1, 1)
A2IAFFM	Conv2d (8 layers)	Kernel_size = (1, 1), stride = (1, 1)
Fully connected layer	FC	Input size = 3 × C, output size = N

## Results

In order to evaluate the effectiveness of the proposed ACAS2F2N in hyperspectral classification tasks, this paper shows experimental comparison and analysis. Specifically, this paper mainly focuses on dataset and experimental parameter settings, baseline comparison algorithm, algorithm performance comparison, ablation analysis, algorithm performance and complexity analysis.

### Dataset and experimental parameter settings

Indian Pines (IP) was collected by the Airborne Visual Infrared Imaging Spectrometer (AVIRIS) in 1992. IP contains a total of 220 continuous bands with a wavelength range of 0.4–2.5 μm. Among them, 20 invalid bands are not considered as research objects, so the experiment contains a total of 200 valid bands. The size of the data is 145 × 145.

The Botswana data set contains a total of 14 categories with a resolution of $1476\times 256$ and a total of 145 effective frequency bands.

Kennedy Space Center (KSC) acquisition is based on AVIRIS. A total of 176 available bands are included, and the wavelength range is 0.4 to 2.5 μm. The size of the data is $512\times 614$. Spatial resolution is 18 m.

The experiment was performed on tesla V100, and the capacity of the graphics card was 16G. The evaluation indicators are overall accuracy (OA), average accuracy (AA), and kappa coefficient (Kappa). During the experiment, on the three datasets, the ratio of training samples, verification samples and test samples is 3%:3%:94%.

### Baseline selection

In order to verify the classification accuracy and real-time performance of the proposed ACAS2F2N, this paper compares the performance of 7 baselines. The specific description is as follows: A2S2KResNet^4^ (TGRS, 2020), DBDA^13^ (RS, 2020), PyResNet^18^ (TGRS2019), DBMA^76^ (RS, 2019), SSRN^14^ (TGRS, 2018), FDSSC^15^ (RS, 2018), ContextNet^17^ (TIP, 2017).

### Algorithm performance comparison

**Figure 5:**
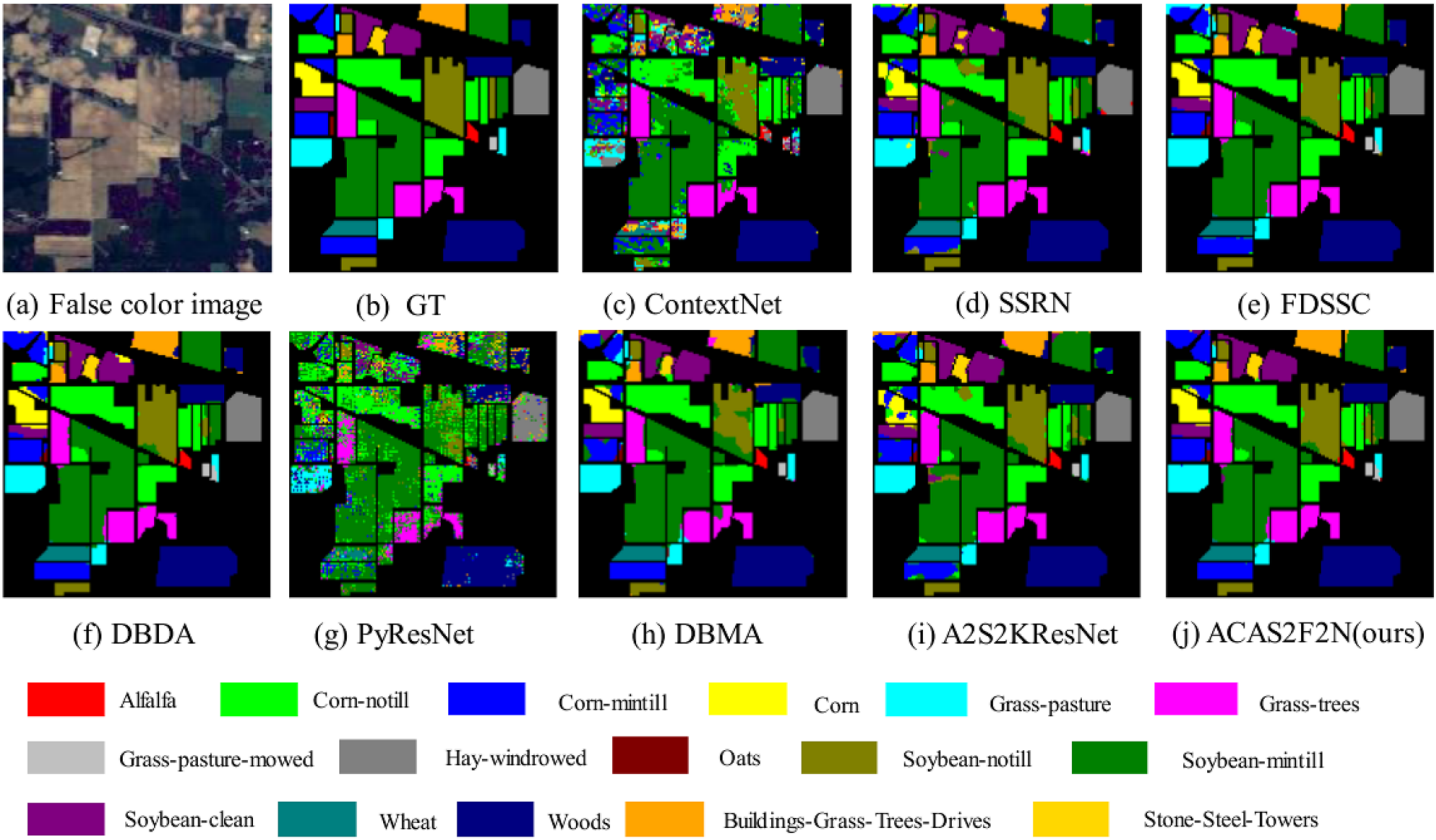
Hyperspectral classification maps on IP. (**a**) False color image. (**b**) Ground truth. (**c**–**i**) Performance of all baselines. (**j**) Performance of the proposed ACAS2F2N. The other content is color and label information.

In order to evaluate algorithm performance fairly, all algorithms maintain the same parameter settings. On the IP dataset, 3% of the data is used for training. The epoch is 200, the code is run 3 times, and the length of the patch is 4. The experimental results on IP are shown in Table 2 and Fig. 5. It can be seen from Table 2 and Fig. 5 that the performance of the proposed ACAS2F2N algorithm is better than other baselines, and the time complexity of the proposed ACAS2F2N is lower. Therefore, it can show high classification accuracy and low time complexity.

**Table 2:** The objective indicators on IP dataset (using 3% training, 3% verification, 94% testing).

Class	Training	Test	ContextNet	SSRN	FDSSC	DBDA	PyResNet	DBMA	A2S2KResNet	Ours
1	3	43	48.57 ± 0.107	94.20 ± 0.08	95.49 ± 0.03	94.26 ± 0.04	37.61 ± 0.15	97.83 ± 0.03	86.35 ± 0.07	94.04 ± 0.03
2	42	1337	76.51 ± 0.04	91.17 ± 0.02	97.78 ± 0.01	94.91 ± 0.04	40.70 ± 0.10	95.12 ± 0.02	92.94 ± 0.03	95.61 ± 0.02
3	24	777	53.93 ± 0.11	90.05 ± 0.09	95.65 ± 0.02	92.00 ± 0.04	38.94 ± 0.03	97.31 ± 0.02	90.78 ± 0.03	95.65 ± 0.02
4	7	224	44.46 ± 0.30	91.81 ± 0.08	93.67 ± 0.04	89.22 ± 0.10	28.95 ± 0.18	90.74 ± 0.05	90.52 ± 0.09	94.83 ± 0.03
5	14	455	70.28 ± 0.12	96.96 ± 0.01	94.03 ± 0.08	96.93 ± 0.01	67.95 ± 0.10	96.50 ± 0.04	99.08 ± 0.01	99.15 ± 0.08
6	21	689	91.90 ± 0.03	98.13 ± 0.00	96.48 ± 0.01	97.49 ± 0.01	63.44 ± 0.11	97.26 ± 0.02	95.73 ± 0.03	97.06 ± 0.02
7	3	25	26.51 ± 0.05	66.67 ± 0.47	66.35 ± 0.09	70.90 ± 0.01	52.56 ± 0.41	86.48 ± 0.05	86.04 ± 0.20	65.64 ± 0.11
8	14	447	85.27 ± 0.13	96.95 ± 0.01	99.35 ± 0.01	100.0 ± 0.21	61.26 ± 0.43	100.0 ± 0.00	97.81 ± 0.01	100.0 ± 0.00
9	3	16	13.70 ± 0.08	23.81 ± 0.34	54.89 ± 0.15	69.21 ± 0.17	14.44 ± 0.14	62.89 ± 0.23	58.06 ± 0.13	66.18 ± 0.01
10	29	918	81.39 $\pm$ 0.02	73.56 $\pm$ 0.06	88.82 $\pm$ 0.08	93.13 $\pm$ 0.03	48.30 $\pm$ 0.18	92.37 $\pm$ 0.05	86.54 $\pm$ 0.03	92.09 $\pm$ 0.03
11	73	2313	79.20 $\pm$ 0.03	95.35 $\pm$ 0.03	97.69 $\pm$ 0.01	96.03 $\pm$ 0.00	53.19 $\pm$ 0.12	92.37 $\pm$ 0.06	91.19 $\pm$ 0.02	96.75 $\pm$ 0.01
12	17	563	51.12 $\pm$ 0.07	89.64 $\pm$ 0.03	98.12 $\pm$ 0.01	96.63 $\pm$ 0.01	52.86 $\pm$ 0.05	94.43 $\pm$ 0.03	94.30 $\pm$ 0.04	95.57 $\pm$ 0.02
13	6	193	68.89 $\pm$ 0.06	94.43 $\pm$ 0.04	92.94 $\pm$ 0.02	98.90 $\pm$ 0.01	44.91 $\pm$ 0.32	99.66 $\pm$ 0.00	97.01 $\pm$ 0.01	97.77 $\pm$ 0.01
14	37	1184	90.52 $\pm$ 0.02	93.78 $\pm$ 0.02	96.94 $\pm$ 0.03	96.24 $\pm$ 0.02	78.18 $\pm$ 0.09	96.81 $\pm$ 0.02	96.47 $\pm$ 0.02	97.72 $\pm$ 0.02
15	11	367	53.88 $\pm$ 0.13	87.03 $\pm$ 0.13	96.94 $\pm$ 0.01	96.01 $\pm$ 0.02	45.10 $\pm$ 0.23	94.88 $\pm$ 0.01	86.18 $\pm$ 0.09	95.21 $\pm$ 0.01
16	3	84	38.73 $\pm$ 0.20	66.67 $\pm$ 0.47	81.45 $\pm$ 0.20	97.86 $\pm$ 0.01	45.45 $\pm$ 0.41	94.21 $\pm$ 0.07	94.69 $\pm$ 0.02	93.13 $\pm$ 0.06
OA (%)	307	9635	76.08 $\pm$ 0.03	90.70 $\pm$ 0.01	95.43 $\pm$ 0.01	95.28 $\pm$ 0.01	52.80 $\pm$ 0.11	94.48 $\pm$ 0.02	92.34 $\pm$ 0.00	96.02 $\pm$ 0.00
AA (%)			60.93 $\pm$ 0.06	84.38 $\pm$ 0.08	90.41 $\pm$ 0.02	92.48 $\pm$ 0.02	48.37 $\pm$ 0.17	93.05 $\pm$ 0.01	90.23 $\pm$ 0.03	92.28 $\pm$ 0.01
Kappa			0.7241 $\pm$ 0.04	0.8941 $\pm$ 0.02	0.9479 $\pm$ 0.01	0.9461 $\pm$ 0.01	0.4427 $\pm$ 0.14	0.9368 $\pm$ 0.02	0.9125 $\pm$ 0.00	0.9546 $\pm$ 0.01
Training time			166.24	236.73	578.67	338.06	507.81	252.64	233.65	99.40
Test time			49.29	37.21	23.93	26.77	103.94	22.34	24.25	20.45

Specifically, in Table 2, A2S2KResNet is proposed in TGRS 2020. The OA, AA, Kappa, training time and test time of A2S2KResNet are 92.34, 90.23, 0.9125, 233.65 and 24.25, respectively. The OA, AA, Kappa, training time, and test time of the proposed ACAS2F2N are 96.02, 92.28, 0.9546, 99.40, and 20.45, respectively. Therefore, compared with A2S2KResNet, the proposed ACAS2F2N OA increased by 3.99%, AA increased by 2.27%, and Kappa increased by 4.61%. In terms of time complexity, the training time was reduced from the original 233.65 s to 99.40 s, and the test time was reduced from the original 24.25 s to 20.45 s. Therefore, the proposed ACAS2F2N has faster convergence. Compared with other baselines, proposed ACAS2F2N can accurately capture position information and enhance the receptive field. In addition, the proposed algorithm uses strip pooling to mine regional information. Proposed ACAS2F2N achieves better classification performance than all the baselines in Table 2 when using 3% data to train the network. In Fig. 5, classification map of the proposed ACAS2F2N is closer to Ground truth (GT), so the experiment verifies the effectiveness of ACAS2F2N on IP.

**Figure 6:**
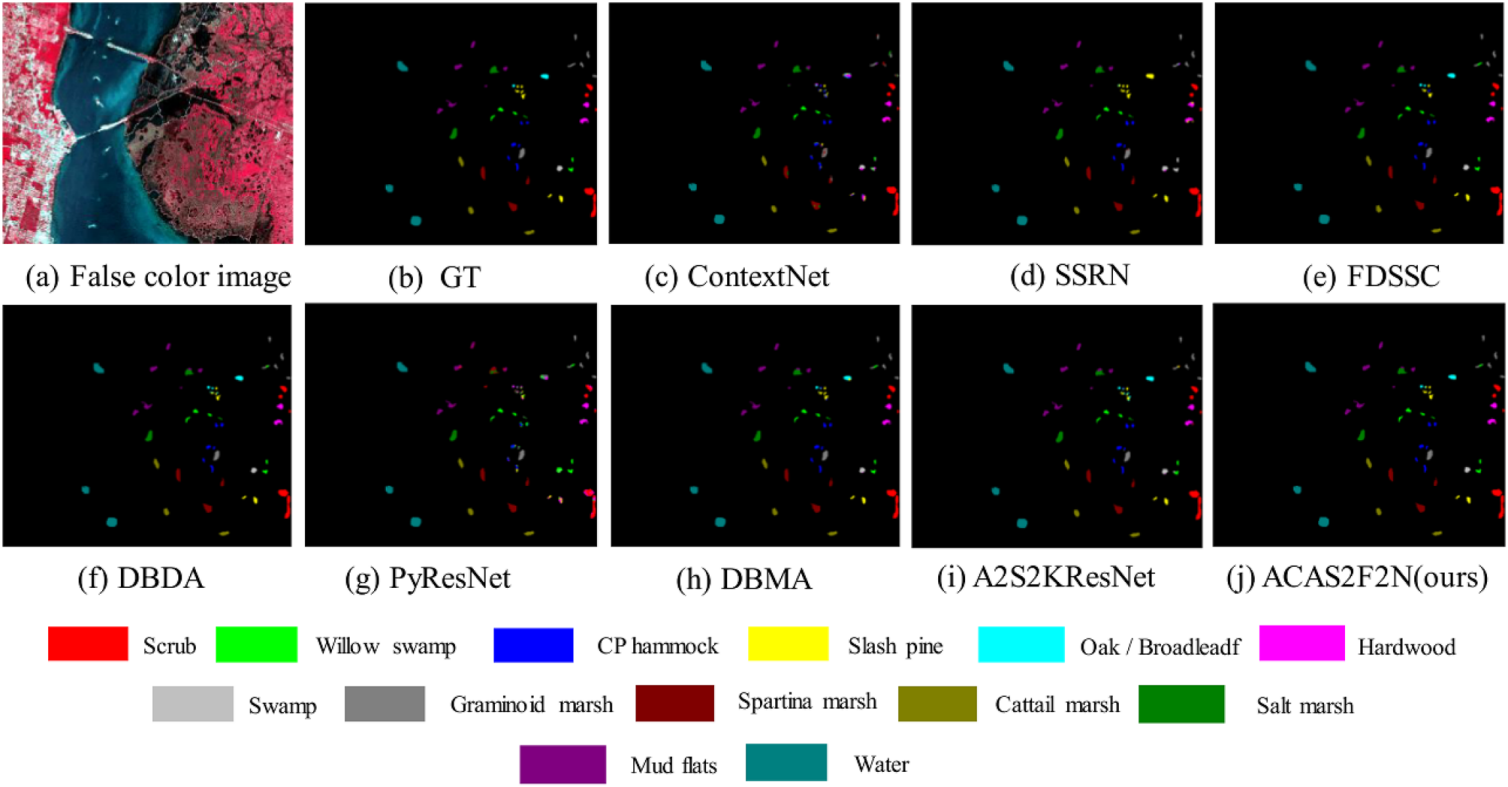
Hyperspectral classification maps on KSC. (**a**) False color image. (**b**) Ground truth. (**c**–**i**) Performance of all baselines. (**j**) Performance of the proposed ACAS2F2N. The other content is color and label information.

On the KSC dataset, Table 3 and Fig. 6 respectively show the objective and subjective hyperspectral classification results. The OA, AA, Kappa, training time and test time of A2S2KResNet are 97.30, 95.83, 0.9699, 305.02 and 18.5, respectively. The OA, AA, Kappa, training time, and test time of the proposed ACAS2F2N are 98.86, 98.44, 0.9873, 117.55, and 15.80, respectively. Therefore, compared with A2S2KResNet, the proposed ACAS2F2N OA increased by 1.60%, AA increased by 2.72%, and Kappa increased by 1.79%. In terms of time complexity, the training time was reduced from the original 305.02 s to 117.55 s, and the test time was reduced from the original 18.5 s to 15.8 s. Therefore, the proposed ACAS2F2N has faster convergence on KSC.

**Table 3:** The objective indicators on KSC dataset (using 3% training, 3% verification, 94% testing).

Class	Training	Test	ContextNet	SSRN	FDSSC	DBDA	PyResNet	DBMA	A2S2KResNet	Ours
1	22	709	98.25 $\pm$ 0.00	99.58 $\pm$ 0.01	99.86 $\pm$ 0.00	100.0 $\pm$ 0.00	87.82 $\pm$ 0.10	99.95 $\pm$ 0.00	99.63 $\pm$ 0.00	99.86 $\pm$ 0.00
2	7	232	79.70 $\pm$ 0.12	83.42 $\pm$ 0.12	96.40 $\pm$ 0.05	97.71 $\pm$ 0.03	69.57 $\pm$ 0.21	96.74 $\pm$ 0.05	98.22 $\pm$ 0.02	97.10 $\pm$ 0.04
3	7	242	63.18 $\pm$ 0.05	85.60 $\pm$ 0.11	93.23 $\pm$ 0.05	99.58 $\pm$ 0.01	76.57 $\pm$ 0.18	90.89 $\pm$ 0.03	87.61 $\pm$ 0.08	92.41 $\pm$ 0.07
4	7	237	64.27 $\pm$ 0.00	71.03 $\pm$ 0.13	95.10 $\pm$ 0.06	94.52 $\pm$ 0.07	74.70 $\pm$ 0.19	85.43 $\pm$ 0.09	97.24 $\pm$ 0.03	98.99 $\pm$ 0.01
5	4	156	60.91 $\pm$ 0.07	78.38 $\pm$ 0.21	88.38 $\pm$ 0.09	84.38 $\pm$ 0.11	45.84 $\pm$ 0.32	95.58 $\pm$ 0.04	83.79 $\pm$ 0.12	94.76 $\pm$ 0.04
6	6	212	73.22 $\pm$ 0.19	88.09 $\pm$ 0.06	94.82 $\pm$ 0.07	96.47 $\pm$ 0.03	64.04 $\pm$ 0.17	98.94 $\pm$ 0.01	92.12 $\pm$ 0.06	99.69 $\pm$ 0.00
7	3	99	60.11 $\pm$ 0.10	50.57 $\pm$ 0.41	88.96 $\pm$ 0.16	97.46 $\pm$ 0.04	57.17 $\pm$ 0.33	93.72 $\pm$ 0.09	91.93 $\pm$ 0.06	100.0 $\pm$ 0.00
8	12	410	95.68 $\pm$ 0.05	99.83 $\pm$ 0.00	100.0 $\pm$ 0.00	98.34 $\pm$ 0.02	95.14 $\pm$ 0.02	99.59 $\pm$ 0.01	99.52 $\pm$ 0.00	99.67 $\pm$ 0.00
9	15	491	93.77 $\pm$ 0.07	97.19 $\pm$ 0.02	100.0 $\pm$ 0.00	100.0 $\pm$ 0.00	85.87 $\pm$ 0.06	99.93 $\pm$ 0.00	99.46 $\pm$ 0.01	99.86 $\pm$ 0.00
10	12	385	100.0 $\pm$ 0.00	100.0 $\pm$ 0.00	100.0 $\pm$ 0.00	99.91 $\pm$ 0.00	99.74 $\pm$ 0.00	100.0 $\pm$ 0.00	100.0 $\pm$ 0.00	100.0 $\pm$ 0.00
11	12	399	94.07 $\pm$ 0.08	97.98 $\pm$ 0.03	100.0 $\pm$ 0.00	100.0 $\pm$ 0.00	100.0 $\pm$ 0.00	100.0 $\pm$ 0.00	100.0 $\pm$ 0.00	100.0 $\pm$ 0.00
12	15	470	98.57 $\pm$ 0.02	95.31 $\pm$ 0.01	98.28 $\pm$ 0.01	95.42 $\pm$ 0.01	96.67 $\pm$ 0.01	94.65 $\pm$ 0.02	96.29 $\pm$ 0.03	97.34 $\pm$ 0.01
13	27	871	99.96 $\pm$ 0.00	99.96 $\pm$ 0.00	100.0 $\pm$ 0.00	100.0 $\pm$ 0.00	94.03 $\pm$ 0.07	100.0 $\pm$ 0.00	100.0 $\pm$ 0.00	100.0 $\pm$ 0.00
OA (%)	149	4913	90.08 $\pm$ 0.02	93.91 $\pm$ 0.01	97.97 $\pm$ 0.00	98.13 $\pm$ 0.00	85.83 $\pm$ 0.03	97.60 $\pm$ 0.00	97.30 $\pm$ 0.00	98.86 $\pm$ 0.00
AA (%)			83.21 $\pm$ 0.01	88.22 $\pm$ 0.04	96.54 $\pm$ 0.01	97.21 $\pm$ 0.00	80.55 $\pm$ 0.04	96.57 $\pm$ 0.01	95.83 $\pm$ 0.00	98.44 $\pm$ 0.00
Kappa			0.8896 $\pm$ 0.02	0.9322 $\pm$ 0.01	0.9774 $\pm$ 0.00	0.9792 $\pm$ 0.00	0.8419 $\pm$ 0.03	0.9733 $\pm$ 0.00	0.9699 $\pm$ 0.00	0.9873 $\pm$ 0.00
Training time			129.90	282.96	774.31	451.19	251.09	433.50	305.02	117.55
Test time			34.03	24.18	28.87	40.90	101.62	49.63	18.50	15.80

**Figure 7:**
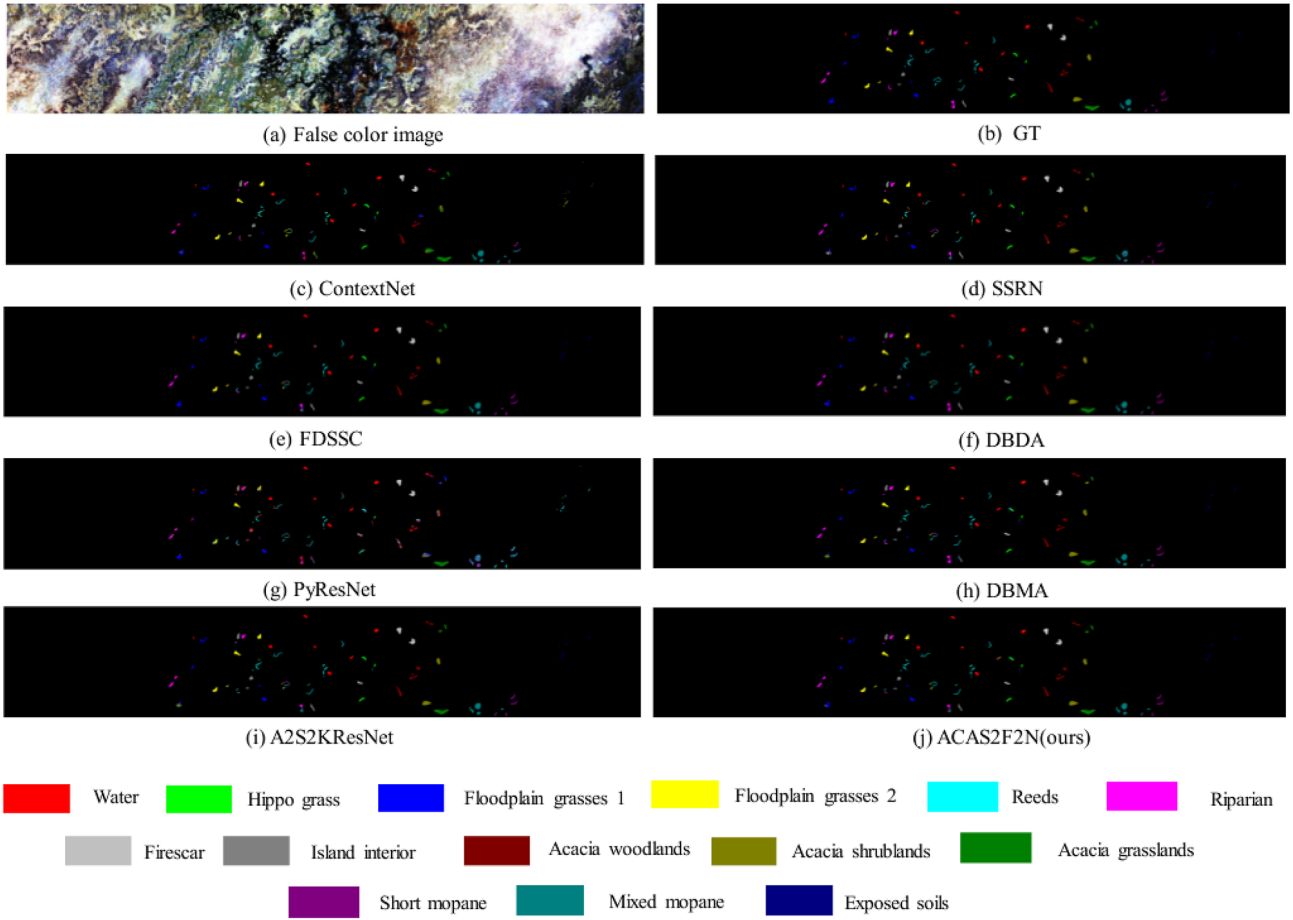
Hyperspectral classification maps on Botswana. (**a**) False color image. (**b**) Ground truth. (**c**–**i**) Performance of all baselines. (**j**) Performance of the proposed ACAS2F2N. The other content is color and label information.

On the Botswana dataset, Table 4 and Fig. 7 respectively show the objective and subjective hyperspectral classification results. A2S2KResNet was proposed and published in TGRS2020. The OA, AA, Kappa, training time and test time of A2S2KResNet are 91.62, 93.37, 0.9091, 47.53, and 8.92, respectively. The OA, AA, Kappa, training time, and test time of the proposed ACAS2F2N are 94.63, 95.72, 0.9418, 50.29, and 7.09, respectively. Therefore, compared with A2S2KResNet, the proposed ACAS2F2N OA increased by 3.28%, AA increased by 2.52%, and Kappa increased by 3.60%. In terms of time complexity, the test time was reduced from the original 8.92 s to 7.09 s. Therefore, the proposed ACAS2F2N has faster convergence on the Botswana dataset.

**Table 4:** The objective indicators on Botswana (using 3% training, 3% verification, 94% testing).

Class	Training	Test	ContextNet	SSRN	FDSSC	DBDA	PyResNet	DBMA	A2S2KResNet	Ours
1	8	254	98.97 $\pm$ 0.01	88.88 $\pm$ 0.08	98.01 $\pm$ 0.02	98.45 $\pm$ 0.01	63.78 $\pm$ 0.22	95.56 $\pm$ 0.03	97.21 $\pm$ 0.01	94.36 $\pm$ 0.07
2	3	97	76.57 $\pm$ 0.08	93.47 $\pm$ 0.05	100.0 $\pm$ 0.00	100.0 $\pm$ 0.00	91.68 $\pm$ 0.07	98.99 $\pm$ 0.01	98.98 $\pm$ 0.01	97.89 $\pm$ 0.01
3	7	243	92.27 $\pm$ 0.05	99.18 $\pm$ 0.01	100.0 $\pm$ 0.00	100.0 $\pm$ 0.00	60.56 $\pm$ 0.24	98.67 $\pm$ 0.01	97.70 $\pm$ 0.03	99.56 $\pm$ 0.01
4	6	203	89.22 $\pm$ 0.09	83.11 $\pm$ 0.04	92.16 $\pm$ 0.02	95.42 $\pm$ 0.04	94.79 $\pm$ 0.06	93.76 $\pm$ 0.03	94.14 $\pm$ 0.03	89.91 $\pm$ 0.06
5	8	254	73.97 $\pm$ 0.16	95.77 $\pm$ 0.04	83.24 $\pm$ 0.07	89.49 $\pm$ 0.02	41.43 $\pm$ 0.13	89.09 $\pm$ 0.06	85.11 $\pm$ 0.05	87.79 $\pm$ 0.06
6	8	253	87.75 $\pm$ 0.05	87.39 $\pm$ 0.04	90.05 $\pm$ 0.09	96.64 $\pm$ 0.03	48.55 $\pm$ 0.12	92.64 $\pm$ 0.04	76.53 $\pm$ 0.09	98.59 $\pm$ 0.01
7	7	248	96.63 $\pm$ 0.04	98.93 $\pm$ 0.01	100.0 $\pm$ 0.00	100.0 $\pm$ 0.00	94.09 $\pm$ 0.01	99.86 $\pm$ 0.00	97.98 $\pm$ 0.01	99.87 $\pm$ 0.00
8	6	193	95.07 $\pm$ 0.01	93.53 $\pm$ 0.09	99.82 $\pm$ 0.00	98.63 $\pm$ 0.01	86.25 $\pm$ 0.14	94.48 $\pm$ 0.05	96.14 $\pm$ 0.03	98.26 $\pm$ 0.02
9	9	297	84.62 $\pm$ 0.12	81.94 $\pm$ 0.09	95.96 $\pm$ 0.03	99.56 $\pm$ 0.01	78.90 $\pm$ 0.10	97.86 $\pm$ 0.02	86.06 $\pm$ 0.06	89.49 $\pm$ 0.08
10	7	229	78.91 $\pm$ 0.22	84.19 $\pm$ 0.11	99.31 $\pm$ 0.01	98.91 $\pm$ 0.02	85.99 $\pm$ 0.03	91.42 $\pm$ 0.10	93.73 $\pm$ 0.08	97.62 $\pm$ 0.03
11	9	283	78.68 $\pm$ 0.14	96.16 $\pm$ 0.04	100.0 $\pm$ 0.00	100.0 $\pm$ 0.00	89.11 $\pm$ 0.06	96.74 $\pm$ 0.03	97.18 $\pm$ 0.02	100.0 $\pm$ 0.00
12	5	166	81.34 $\pm$ 0.26	90.40 $\pm$ 0.07	98.60 $\pm$ 0.01	99.46 $\pm$ 0.01	88.23 $\pm$ 0.06	98.10 $\pm$ 0.03	98.90 $\pm$ 0.01	100.0 $\pm$ 0.00
13	8	252	68.08 $\pm$ 0.06	84.86 $\pm$ 0.08	90.83 $\pm$ 0.07	90.77 $\pm$ 0.07	77.68 $\pm$ 0.09	88.53 $\pm$ 0.08	87.47 $\pm$ 0.10	86.70 $\pm$ 0.09
14	3	90	88.52 $\pm$ 0.16	98.43 $\pm$ 0.02	100.0 $\pm$ 0.00	100.0 $\pm$ 0.00	100.0 $\pm$ 0.00	100.0 $\pm$ 0.00	100.0 $\pm$ 0.00	100.0 $\pm$ 0.00
OA (%)	94	3060	82.57 $\pm$ 0.03	89.19 $\pm$ 0.01	95.47 $\pm$ 0.01	97.25 $\pm$ 0.01	63.05 $\pm$ 0.06	94.55 $\pm$ 0.02	91.62 $\pm$ 0.01	94.63 $\pm$ 0.00
AA (%)			85.04 $\pm$ 0.01	91.16 $\pm$ 0.01	96.28 $\pm$ 0.01	97.67 $\pm$ 0.01	78.65 $\pm$ 0.03	95.41 $\pm$ 0.01	93.37 $\pm$ 0.01	95.72 $\pm$ 0.00
Kappa			0.8107 $\pm$ 0.04	0.8829 $\pm$ 0.01	0.9509 $\pm$ 0.01	0.9702 $\pm$ 0.01	0.5981 $\pm$ 0.07	0.9409 $\pm$ 0.02	0.9091 $\pm$ 0.01	0.9418 $\pm$ 0.00
Training time			40.53	90.40	179.32	146.67	120.34	72.09	47.53	50.29
Test time			10.74	8.88	8.82	10.85	22.96	6.75	8.92	7.09

In Table 4, among the 7 baseline algorithms, the performance of DBDA and FDSSC algorithms (OA, AA and Kappa) is better than the performance of the proposed ACAS2F2N algorithm. However, the model complexity of DBDA and FDSSC algorithm is higher, and the model time consumption is much greater than the proposed ACAS2F2N algorithm. Comprehensively comparing Tables 2 and 3, the performance of the proposed algorithm is better than that of DBDA and FDSSC.

#### Algorithm performance comparison summary

Tables 2, 3 and 4 are comparisons of objective indicators of algorithms (OA, AA, Kappa, training time, and test time) on the three datasets (IP, KSC and Botswana). Figures 5, 6 and 7 are the results of algorithm classification maps, which respectively illustrate the results of hyperspectral classification and classification color labels. The experiment selects 7 baseline algorithms for performance comparison, of which 7 baselines are A2S2KResNet^4^ (TGRS, 2020), DBDA^13^ (RS, 2020), PyResNet^18^ (TGRS2019), DBMA^76^ (RS, 2019), SSRN^14^ (TGRS, 2018), FDSSC^15^ (RS, 2018), and ContextNet^17^ (TIP, 2017). Experimental results verify the effectiveness and real-time performance of the proposed ACAS2F2N. The proposed ACAS2F2N has lower model complexity while improving the performance of hyperspectral classification.

In terms of specific indicators (OA, AA, Kappa), compared to A2S2KResNet (TGRS 2020), the proposed ACAS2F2N OA increased by 3.99%, AA increased by 2.27%, and Kappa increased by 4.61% on IP dataset; the proposed ACAS2F2N OA increased by 1.60%, AA increased by 2.72%, and Kappa increased by 1.79% on KSC dataset; the proposed ACAS2F2N OA increased by 3.28%, AA increased by 2.52%, and Kappa increased by 3.60% on Botswana dataset.

In terms of model complexity, compared to A2S2KResNet (TGRS 2020), the training time was reduced from the original 233.65 s to 99.40 s, and the test time was reduced from the original 24.25 s to 20.45 s on IP dataset; the training time was reduced from the original 305.02 s to 117.55 s, and the test time was reduced from the original 18.5 s to 15.8 s on KSC dataset; the training time was increased from the original 47.53 s to 50.29 s, and the test time was reduced from the original 8.92 s to 7.09 s on Botswana dataset.

### Ablation analysis

Next, this paper shows the ablation analysis of the algorithm. Among them, Table 5 is the ablation analysis on the IP data set, Table 6 is the ablation analysis on the KSC data set, and Table 7 is the ablation analysis on the Botswana data set. For the convenience of description, coordinate attention module is abbreviated as CAM, strip pooling module is abbreviated as SPM, and Asymmetric adaptive iterative attention feature fusion module is abbreviated as A2IAFFM.

**Table 5:** Ablation analysis on IP dataset.

Network module			Feature fusion method		Algorithm performance		
CAM	SPM	A2IAFFM	Concat	Add	OA (%)	AA (%)	Kappa
√	√	√	√		96.02	92.28	0.9546
√		√	√		93.23	89.05	0.9229
	√	√	√		93.39	89.43	0.9246
√	√	√		√	93.26	89.63	0.9232
√					92.80	88.37	0.9180
	√				93.31	89.91	0.9238

**Table 6:** Ablation analysis on KSC dataset.

Network module			Feature fusion method		Algorithm performance		
CAM	SPM	A2IAFFM	Concat	Add	OA (%)	AA (%)	Kappa
√	√	√	√		98.86	98.44	0.9873
√		√	√		97.99	98.28	0.9777
	√	√	√		97.63	96.78	0.9736
√	√	√		√	97.52	97.05	0.9724
√					97.60	96.87	0.9332
	√				95.60	95.57	0.9509

**Table 7:** Ablation analysis on Botswana dataset.

Network module			Feature fusion method		Algorithm performance		
CAM	SPM	A2IAFFM	Concat	Add	OA (%)	AA (%)	Kappa
√	√	√	√		94.63	95.72	0.9418
√		√	√		90.21	92.19	0.8938
	√	√	√		89.92	92.08	0.8907
√	√	√		√	89.51	91.81	0.8863
√					89.53	91.49	0.8865
	√				89.72	91.82	0.8886

The overall result of the algorithm in this paper is shown in Fig. 1. The network modules of ablation analysis include CAM, SPM and A2IAFFM. Feature fusion methods include concat and add operations. If the proposed algorithm removes the A2IAFFM module, then the algorithm only includes CAM or SPM network blocks, and the algorithm does not include feature fusion methods. The ablation analysis experiment is shown in Tables 5, 6 and 7. The experimental results show that the mentioned modules (CAM, SPM and A2IAFFM) all have a positive effect on the hyperspectral classification. In this paper, Concat is used to fuse the features of the three modules. At this time, the algorithm in this paper can experiment with the best hyperspectral classification performance.

### The impact of training size

**Figure 8:**
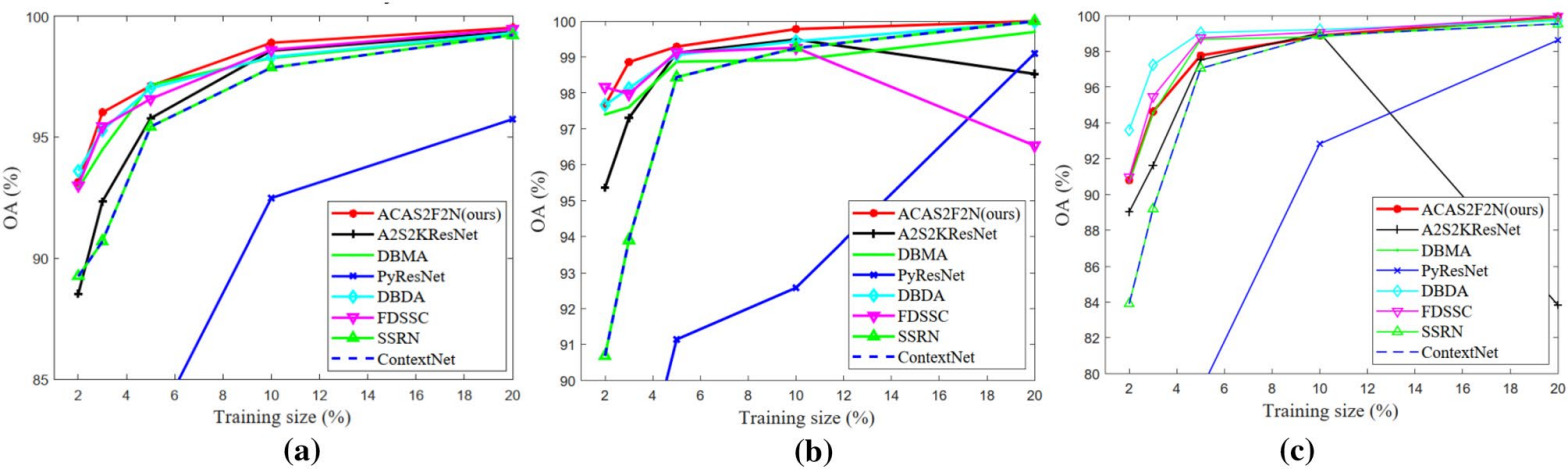
OA performance under different training sizes. (**a**) IP dataset. (**b**) KSC dataset. (**c**) Botswana dataset.

**Figure 9:**
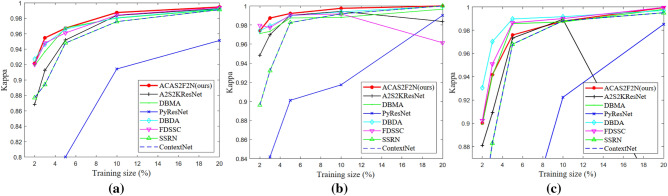
Kappa performance under different training sizes. (**a**) IP dataset. (**b**) KSC dataset. (**c**) Botswana dataset.

The number of training samples has a great influence for hyperspectral classification performance. Therefore, we analyze the impact of training size on OA, Kappa, Training time and test time. Figure 8 shows the OA results of different algorithms under different training sizes. As the training size increases, the OA of the proposed ACAS2F2N also increases. On the IP and KSC datasets, as the training size changes, the proposed ACAS2F2N both show the best performance. On the Botswana dataset, as the training size increases, the proposed ACAS2F2N hyperspectral classification accuracy also increases. Although the accuracy of the proposed algorithm is not optimal on the Botswana dataset, the algorithm has the lowest time complexity. In addition, the proposed algorithm is easier to extend. In addition, the proposed algorithm has the best performance on the IP and KSC datasets, and the proposed ACAS2F2N has less time complexity. Figure 9 shows Kappa performance under different training sizes. Figures 9 and 8 have similar conclusions and verify the effectiveness of the proposed ACAS2F2N.

Specifically, On the IP dataset, when the training size is 2%, 3%, 5%, 10%, and 20%, compared to the A2S2KResNet (TGRS 2020) algorithm, the OA performance of the proposed ACAS2F2N is improved by 5.21%, 3.99%, 1.40%, 0.33% and 0.17%, respectively. On the IP dataset, when the training size is 2%, 3%, 5%, 10%, and 20%, compared to the A2S2KResNet, the Kappa performance of the proposed ACAS2F2N is improved by 6.14%, 4.61%, 1.62%, 0.39% and 0.20%, respectively.

On the KSC dataset, when the training size is 2%, 3%, 5%, 10%, and 20%, compared to the A2S2KResNet (TGRS 2020) algorithm, the OA performance of the proposed ACAS2F2N is improved by 2.41%, 1.60%, 0.17%, 0.29% and 1.49%, respectively. On the KSC dataset, when the training size is 2%, 3%, 5%, 10%, and 20%, compared to the A2S2KResNet, the Kappa performance of the proposed ACAS2F2N is improved by 2.71%, 1.79%, 0.19%, 0.32% and 1.66%, respectively.

On the Botswana dataset, when the training size is 2%, 3%, 5%, 10%, and 20%, compared to the A2S2KResNet (TGRS 2020) algorithm, the OA performance of the proposed ACAS2F2N is improved by 1.99%, 3.29%, 0.26%, − 0.15% and 19.24%, respectively. On the Botswana dataset, when the training size is 2%, 3%, 5%, 10%, and 20%, compared to the A2S2KResNet, the Kappa performance of the proposed ACAS2F2N is improved by 2.18%, 3.60%, 0.28%, -0.16% and 20.93% respectively. The experimental results show the retrieval performance of the algorithm under different training sizes, and verify the effectiveness of the proposed ACAS2F2N.

### Time consumption analysis

Time consumption analysis demonstrates the convergence of the algorithm. Time consumption analysis is also a concrete manifestation of model complexity. This article gives the time consumption of all algorithms under different training sizes, including training time and test time.

**Figure 10:**
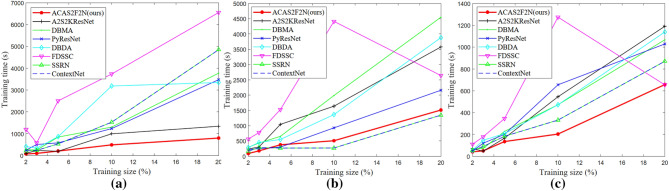
Training time under different training sizes. (**a**) IP dataset. (**b**) KSC dataset. (**c**) Botswana dataset.

**Figure 11:**
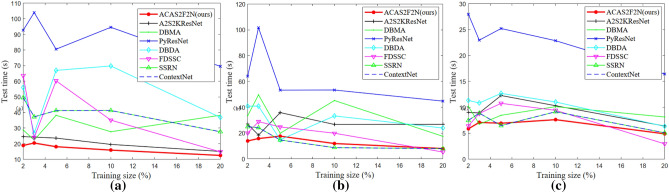
Test time under different training sizes. (**a**) IP dataset. (**b**) KSC dataset. (**c**) Botswana dataset.

Figure 10 shows Training time under different training sizes. Figure 11 shows Test time under different training sizes. Figures 10 and 11 jointly illustrate the time complexity of the proposed algorithm and the baseline. It can be seen from Figs. 10 and 11 that the proposed ACAS2F2N has less time consumption. In Figs. 8 and 9, we illustrate the impact of different training sizes on OA and Kappa. In terms of time complexity and accuracy of hyperspectral classification, the performance of the proposed ACAS2F2N is better than that of the baselines. Experiments verify the effectiveness of the proposed algorithm.

### Training and verification accuracy

**Figure 12:**
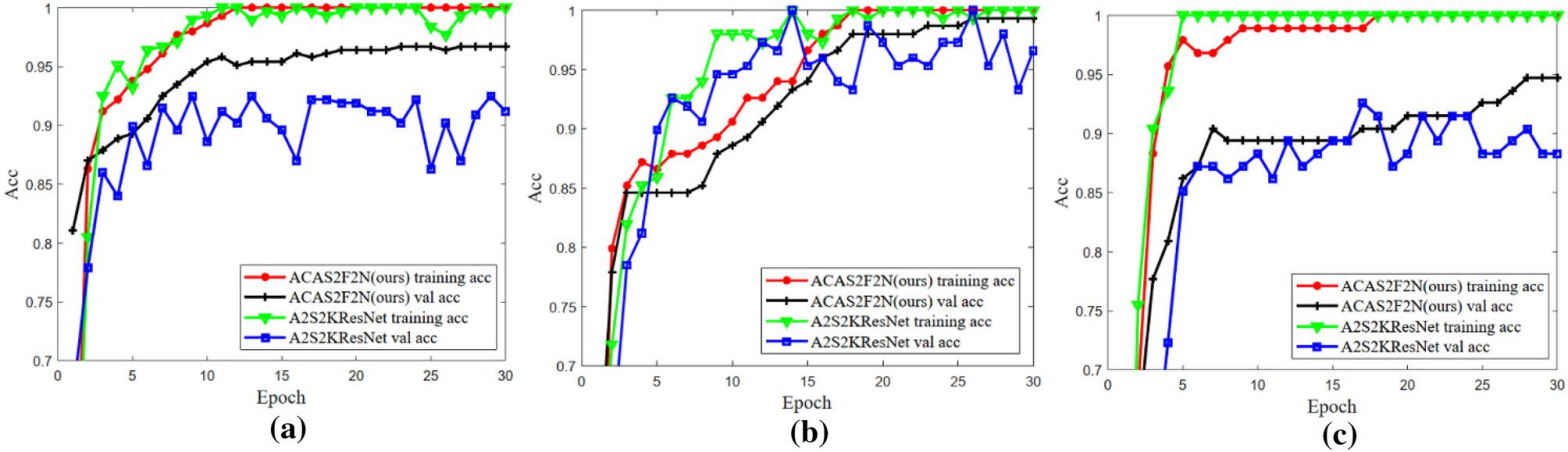
Training and verification accuracy. (**a**) IP dataset. (**b**) KSC dataset. (**c**) Botswana dataset.

Figure 12 shows the training and verification accuracy. Figure 12 shows that the performance of the proposed ACAS2F2N algorithm is better than that of A2S2KResNet. In short, the proposed ACAS2F2N shows better classification performance on three hyperspectral datasets. Achieving high classification accuracy is accompanied by lower time consumption.

## Conclusions

In this paper, we propose an asymmetric coordinate attention spectral-spatial feature fusion network (ACAS2F2N) to complete the hyperspectral classification task. Compared with the baselines, the proposed algorithm can improve the performance of hyperspectral image classification and has lower model complexity. Specifically, Coordinate attention is used to obtain accurate coordinate information and channel relationship. The strip pooling module was introduced to increase the network’s receptive field and avoid irrelevant information brought by conventional convolution kernels. Asymmetric adaptive iterative attention feature fusion module is adopted to extract the discriminative spectral-spatial features. The experimental was performed on three datasets (IP, KSC and Botswana), and the experimental results show that the performance of the proposed ACAS2F2N is better than the baselines. In addition, the proposed ACAS2F2N has low model complexity and time consumption.
